# Cervical Tuberculosis in the Postmenopausal Period: A Case of Mistaken Identity With Carcinoma

**DOI:** 10.7759/cureus.97336

**Published:** 2025-11-20

**Authors:** Nafisa M El Sunni, Sara Adam, Saadia A Noreldeen, Mohamed Y Adam

**Affiliations:** 1 Obstetrics and Gynaecology, Wad Madani Teaching Hospital, Wad Madani, SDN; 2 Medicine and Paediatrics, Ipswich General Hospital, Ipswich, GBR; 3 Women and Children Health, Princess Alexandra Hospital NHS Trust, Harlow, GBR; 4 General Medicine, Sudan International University, Khartoum, SDN

**Keywords:** caseating granuloma, cervical tuberculosis, examination under anesthesia, female genital tuberculosis, postmenopausal discharge

## Abstract

Female genital tuberculosis (FGTB) is an uncommon manifestation of extrapulmonary tuberculosis that can closely resemble gynaecological malignancies, often resulting in delayed diagnosis and treatment. While the fallopian tubes are the commonest organs to be affected, isolated cervical involvement is exceedingly rare. We report the case of a 60-year-old multiparous woman from a tuberculosis-endemic region in Sudan who presented with severe lower abdominal, pelvic, and perineal pain, along with odourless vaginal discharge persisting for four months. Her condition had rendered her bedridden. Clinical examination revealed a nodular cervix, initially suggestive of cervical cancer. However, histopathological analysis of cervical and endometrial biopsies confirmed caseating granulomas indicative of tuberculosis. The patient had no prior history of tuberculosis or malignancy and had never undergone cervical cancer screening. Imaging showed a uterocervical mass without signs of spinal or metastatic disease, and pulmonary tuberculosis was ruled out via chest X-ray and sputum analysis. She was treated with a standard six-month anti-tuberculosis regimen, resulting in significant clinical improvement and restored mobility. This case highlights the importance of considering cervical tuberculosis in the differential diagnosis of postmenopausal women presenting with pelvic symptoms in endemic areas. Early histological diagnosis and timely initiation of therapy are crucial for favourable outcomes.

## Introduction

Tuberculosis (TB) remains one of the leading causes of mortality from infectious disease worldwide, despite extensive global eradication efforts [1]. Female genital tuberculosis (FGTB) is an uncommon form of extrapulmonary TB, with reported incidence varying widely from 0.045% to 24% due to its nonspecific clinical presentation and diagnostic challenges [2-4]. This issue is especially significant in indigenous, rural, and medically disadvantaged areas, where access to advanced diagnostic instruments is restricted and extrapulmonary tuberculosis frequently remains unrecognised.

Female genital tuberculosis (FGTB) is primarily caused by *Mycobacterium tuberculosis*. Mycobacterium can reach the female genital tract through different routes. The first route is haematogenous spread from a primary pulmonary tuberculosis. The second route is through lymphatic dissemination from nearby infected nodes or anywhere in the body. The third route is direct spread from adjacent infected organs, such as the bowel or peritoneum [2]. The fallopian tubes are the most frequently affected organs in FGTB, involved in 90-100% of cases, followed by the endometrium (50-80%), ovaries (20-30%), and cervix (5-15%) [5].

Although rare, FGTB can cause significant morbidity, infertility, and chronic pelvic pain. Cervical tuberculosis is even more unusual and is often misdiagnosed as cervical carcinoma because of similar clinical features [6].

This report presents a rare case of isolated cervical tuberculosis in a postmenopausal woman. This particular case highlights a significant diagnostic challenge, influenced both by the patient's demographic background and the atypical presentation of female genital tuberculosis (FGTB). It also highlights ongoing discrepancies in tuberculosis detection and treatment, particularly in areas lacking sufficient medical resources, where delayed or overlooked diagnoses are prevalent. In many tuberculosis-endemic areas, healthcare infrastructure is under-resourced and diagnostic facilities are limited. As a result, patients often present at advanced stages of illness, having faced significant delays due to the need to travel long distances to access medical care. This case illustrates such challenges, which are unfortunately typical in this setting. Even in tuberculosis-endemic regions, the diagnosis of cervical tuberculosis is rarely considered due to its extreme rarity. 

## Case presentation

A 60-year-old multiparous woman presented to the obstetrics and gynaecology emergency department, brought in a wheelchair by her son. She had been referred from a rural hospital. The patient complained of severe pelvic and perineal pain, accompanied by vaginal discharge. The pain was progressive and severe, leaving the patient bedridden. Previously, she was independent. She had previously been in good health and physically active, regularly performing household chores and managing farm work. Over four months, the pain worsened to the point that even minimal movement became intolerable. The pain was a constant dull ache with sharp exacerbations. It was localised to the lower abdomen, pelvis, and groin, with radiation to the lower back on movement. The pain showed minimal improvement and was only partially relieved by analgesics. 

The patient also observed an odourless, clear vaginal discharge, which preceded the onset of severe pain by approximately two months. She denied postmenopausal bleeding since menopause 20 years earlier. There were no urinary or bowel symptoms. There were no systemic symptoms of malignancy, such as weight loss or anorexia. She denied respiratory symptoms suggestive of pulmonary tuberculosis and had no history of fever, night sweats, or cough. The patient had hypertension, managed with amlodipine 5 mg daily, and a five-year history of lumbar disc prolapse. She had no previous surgeries or hospital admissions. She had never been diagnosed with tuberculosis or undergone pap smear screening. All five pregnancies were uncomplicated vaginal deliveries. She lived in a tuberculosis-endemic region in Sudan and had contact with individuals diagnosed with TB. She was a lifelong non-smoker, married since age 18 to a single partner, and denied alcohol use. There was no family history of malignancy. The patient appeared in pain but was haemodynamically stable, afebrile, and not pale or jaundiced. There was no palpable lymphadenopathy.

Abdominal examination revealed soft, non-tender, with no palpable masses or organomegaly. Pelvic examination revealed perineal tenderness. The vulva was tightly circumcised, the vagina was healthy, and there was a clear, odourless vaginal discharge. The cervix appeared irregular with nodular masses. The adnexa and the pouch of Douglas were free. Rectal mucosa was intact, and pelvic sidewalls were free. Unfortunately, images of the cervix were not done, as colposcopy services are not available in Sudan.

Given the age and clinical presentation, cervical carcinoma was suspected. Staging investigations were scheduled, including a cervical biopsy and examination under anaesthesia (EUA). Complete blood count, liver, and renal function tests were within normal limits. Tumour markers were unremarkable (Table 1).

**Table 1:** Initial investigations all within normal limits. Hb: haemoglobin; WBC: white blood cell; CA-125: cancer antigen 125; ALT: alanine aminotransferase; AST: aspartate aminotransferase.

Parameters	Result	Reference range
Hb	128 g/L	115–155 g/L
WBC	7.2 × 10⁹/L	4.0–11.0 × 10⁹/L
Platelets	251 × 10⁹/L	150–400 × 10⁹/L
CA-125	18 U/mL	<35 U/mL
ALT	27 U/L	10–35 U/L
AST	22 U/L	10–40 U/L
Urea	5.4 mmol/L	2.5–7.8 mmol/L
Creatinine	81 µmol/L	45–90 µmol/L

MRI of the spine (performed preoperatively for anaesthesia safety) revealed no significant disc bulge except mild exit foraminal narrowing, normal conus medullaris position and signal, and normal cauda equina nerve roots. Vertebral alignment and height were normal, with no paraspinal or prevertebral soft tissue abnormalities.

Abdominal and pelvic ultrasound: normal abdominal organs; uterocervical mass measuring 3.3 × 1.8 cm causing uterine outflow obstruction with increased Doppler vascularity; no ascites or lymphadenopathy. The uterus was of normal size with an endometrial thickness of 8 mm. No adnexal mass was seen, and neither of the ovaries was visualised. Planned pelvic MRI and cystoscopy for cervical cancer staging were requested.

EUA revealed a healthy vagina; cervical tissue was friable and nodular. Multiple biopsies were obtained. The uterus was normal in size; curettage was performed, and tissue was sent for histopathology. Macroscopically, the histopathology shows a cervix measuring 0.3 × 0.2 × 0.2 cm with irregular, soft, white tissue, and the endometrium measures 0.4 × 0.3 × 0.2 cm, consisting of dark grey soft tissue aggregates. Microscopically, both biopsies revealed necrotic tissue with caseating tuberculoid granulomas and epithelioid histiocytes. No neoplastic cells were identified.

**Figure 1:**
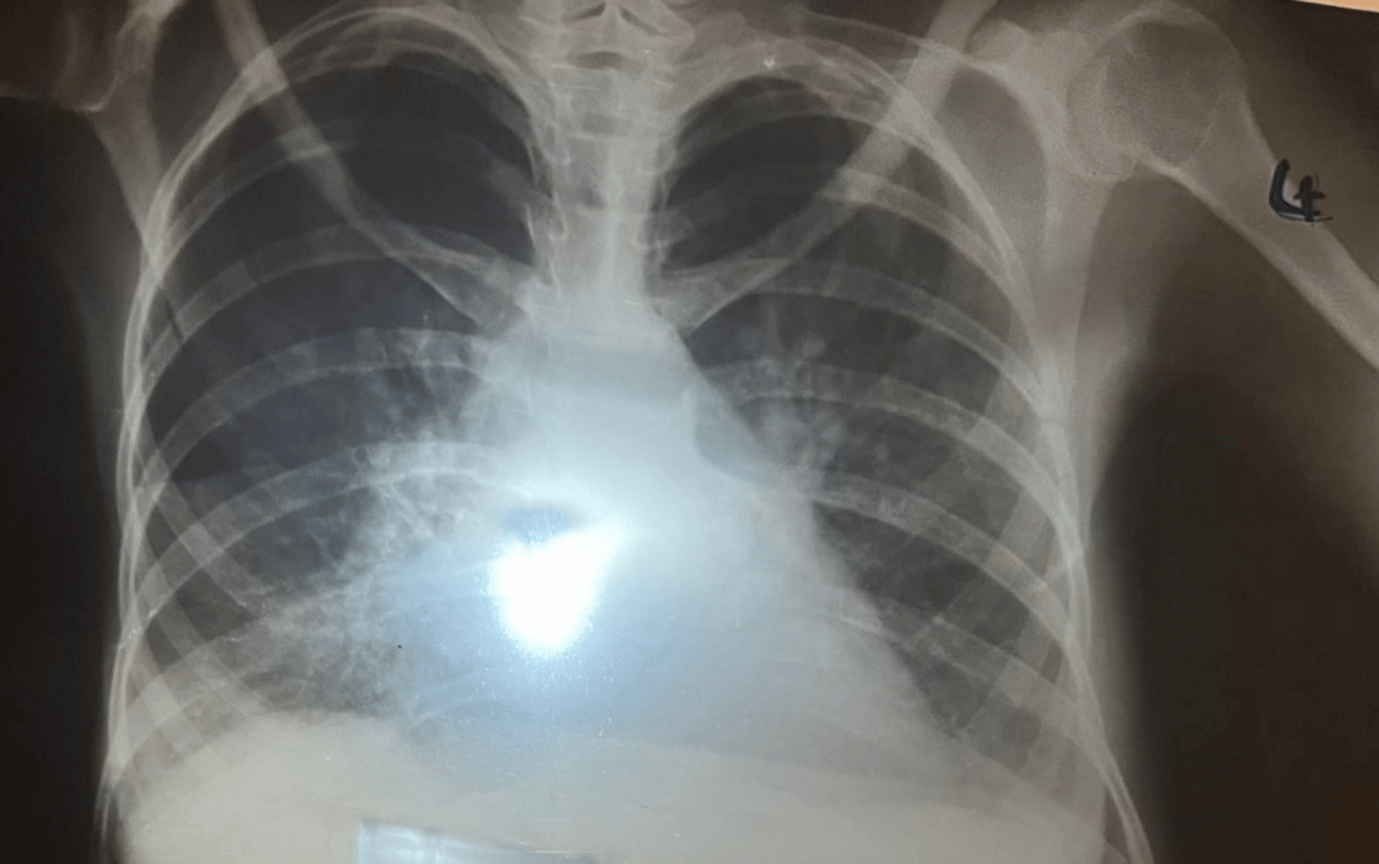
Normal chest x-ray.

Following consultation with the infectious disease control centre, chest X-ray and sputum staining with Ziehl-Neelsen stain were performed. Both were negative (Figure 1).

Further diagnostic evaluation with the GeneXpert assay was arranged to confirm the diagnosis of tuberculosis (TB). A tissue specimen was submitted for analysis at the World Health Organization (WHO)-affiliated laboratory located within the hospital. The test detects DNA sequences specific to *Mycobacterium tuberculosis* and simultaneously assesses rifampicin resistance. The result was positive for TB, with no evidence of rifampicin resistance.

Although GeneXpert offers rapid molecular confirmation of tuberculosis, bacteriological (mycobacterial) culture is the gold standard for complete microbiological analysis and extensive medication susceptibility testing. However, this was not performed in this case due to limited laboratory capacity.

The patient's pelvic pain gradually decreased after starting normal anti-tuberculosis medication, and within a few months, she was able to regain her independence and mobility.

## Discussion

Female genital tuberculosis remains a diagnostic challenge due to its nonspecific presentation and resemblance to malignancy or chronic cervicitis [6,7]. Isolated cervical tuberculosis is especially rare, accounting for less than 1% of genital TB cases, with an estimated prevalence of 0.1-0.65% of all TB cases [8,9]. Several reports describe cases initially misdiagnosed as cervical cancer, leading to unnecessary surgical intervention or delayed anti-tuberculosis therapy [10,11].

Given the patient's age, postmenopausal status, and the existence of a nodular, friable cervix, cervical carcinoma was the primary differential diagnosis in this instance. Nonetheless, several characteristics made it easier to differentiate between cancer and TB. In contrast to carcinoma, cervical tuberculosis usually does not exhibit ulcerative or infiltrative growth patterns and frequently does not manifest with postcoital or postmenopausal bleeding. Histologically, tuberculosis exhibits epithelioid granulomas with Langhans-type giant cells and caseous necrosis, while carcinoma displays dysplastic epithelial cells with stromal invasion. The absence of malignant cells on biopsy, along with the presence of granulomatous inflammation and a positive GeneXpert test for *Mycobacterium tuberculosis*, confirmed the diagnosis of cervical tuberculosis and excluded carcinoma.

Histopathology remains the gold standard for diagnosis, with identification of caseating granulomas or detection of *Mycobacterium tuberculosis* via culture or PCR [12,13]. Due to the paucibacillary nature of FGTB, cultures and Ziehl-Neelsen stains often yield negative results. This highlights the importance of newer molecular diagnostic tools. The GeneXpert assay allows rapid detection of *Mycobacterium tuberculosis* DNA and simultaneous assessment of rifampicin resistance. Several studies support its role in genital TB diagnosis, particularly when histopathology is inconclusive or in low-resource endemic areas [14,15].

The GeneXpert test was used in this case to aid in diagnosing tuberculosis using tissue samples and paraffin-embedded specimens. Despite being situated in a low-resource setting, the hospital benefits from access to advanced diagnostic technologies through the WHO tuberculosis treatment and prevention program. The availability of GeneXpert has proven invaluable, particularly in endemic regions with rising rates of drug-resistant TB. In this case, the test not only facilitated rapid detection of *Mycobacterium tuberculosis* but also provided critical information on rifampicin resistance. This is a TB-endemic area with emerging cases of rifampicin resistance; hence, using the GeneXpert test helps in choosing an appropriate therapeutic regimen.

WHO guidelines suggest that extrapulmonary TB can be diagnosed based on one culture-positive specimen, positive histology, or strong clinical evidence [16]. In this case, there was no clinical suspicion initially, but histopathology and molecular testing were used.

Clinically, cervical TB may present with vaginal discharge, postcoital bleeding, pelvic pain, or ulcerative cervical lesions, often indistinguishable from carcinoma [10]. Our patient's presentation with chronic pelvic pain and discharge, but without systemic or bleeding symptoms, highlights the variability of presentation. This variability further complicates recognition. However, this patient's nodular cervical appearance warranted a biopsy.

Standard medical management with a six-month multidrug regimen remains highly effective, with surgery reserved for complications such as tubo-ovarian abscesses. The patient's rapid symptomatic improvement highlights the efficacy of prompt medical therapy.

This case underscores the importance of considering cervical tuberculosis in postmenopausal women presenting with vaginal discharge or pelvic pain, particularly in TB-endemic regions.

## Conclusions

Isolated cervical tuberculosis is an exceptionally rare form of female genital TB and can closely mimic cervical carcinoma, particularly in postmenopausal women. In endemic regions, clinicians should maintain a high index of suspicion for TB in women presenting with atypical cervical lesions or unexplained pelvic symptoms. Histopathological confirmation remains the cornerstone of diagnosis, while molecular testing, such as GeneXpert, provides rapid detection and drug resistance profiling. Prompt initiation of standard six-month anti-tuberculosis therapy is highly effective and prevents unnecessary surgical intervention.
